# Effects of Different Intervertebral Space Heights on Nerve Root Tension during Posterior Lumbar Interbody Fusion

**DOI:** 10.1111/os.13649

**Published:** 2023-02-27

**Authors:** Yujie Wu, Tong Zhu, Zhiyi Fu

**Affiliations:** ^1^ Shanghai Key Laboratory of Orthopaedic Implants, Department of Orthopaedic Surgery, Shanghai Ninth People's Hospital Shanghai Jiao Tong University School of Medicine Shanghai China

**Keywords:** Intervertebral Space Height, Nerve Root, Posterior Lumbar Interbody Fusion, Tension

## Abstract

**Objective:**

There is no effective standard method to evaluate whether the nerve root tension is restored during lumbar decompression surgery, which is an important indicator for the recovery of nerve function. This study aimed to investigate the feasibility of intraoperative nerve root tension measurement and to confirm the correlation between nerve root tension and intervertebral space height.

**Methods:**

A total of 54 consecutive patients (mean age, 54.3 years; range, 25–68 years) received posterior lumbar interbody fusion (PLIF) for lumbar disc herniation (LDH) with lumbar spinal stenosis and instability. The 110%, 120%, 130%, 140% height values of each lesion were calculated based on preoperative measurements of the intervertebral space height. The heights were intraoperatively expanded after the intervertebral disc was removed using the interbody fusion cage model. The tension value of nerve root was measured by pulling the nerve root for 5 mm with a self‐made measuring device. The nerve root tension value was measured before decompression, after discectomy at 100%, 110%, 120%, 130%, and 140% of the height of each intervertebral space, and after placement of the cage *during intraoperative* nerve root tension monitoring.

**Results:**

The nerve root tension values at 100%, 110%, 120%, and 130% heights were significantly lower than those before decompression, and there was no statistical significance among the four groups. The nerve root tension value was significantly higher at 140% height and was statistically significant compared with that of 130% height. The nerve root tension value after cage placement was significantly lower than that before decompression (1.32 ± 0.22 N vs*.* 0.61 ± 0.17 N, *p* < 0.01), and the postoperative VAS score was also significantly improved (7.0 ± 2.24 vs*.* 0.8 ± 0.84, *p* < 0.01). The nerve root tension was positively correlated with the VAS score (F = 85.19, *p* < 0.01; F = 78.65, *p* < 0.01).

**Conclusion:**

This study demonstrates that nerve root tonometry can perform instant noninvasive intraoperative nerve root tension measurement. There is a correlation between nerve root tension value and VAS score. We found that when the height of the intervertebral space was increased to 140% of the original height, the nerve root tension increased the risk of injury significantly.

## Graphical abstract

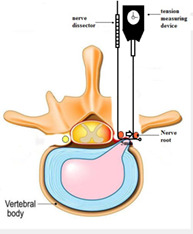

## Introduction

Lumbar radiculopathy is usually caused by a herniated disc, and typically comorbid with sciatica.^1^ The root cause of lumbar radiculopathy patient's radiating pain in the lower extremities is due to the mechanical compression of the lumbar nerve roots increasing its own tension, which triggers a series of ischemia and hypoxia reactions. It has been shown that excessive traction of the nerve root can also cause nerve ischemia injury. This may explain why some patients experience foot drop complications after lumbar posterior lumbar interbody fusion (PLIF) surgery. Bach's results showed more than 10 mm cage heights can lead to the supraphysiological intervertebral space repairing and potential complications.^2^

At present, in *lumbar* spinal *decompression* (LSD) *and lumbar discectomy* (LD) surgery. Clinicians can completely relieve the physical compression of the nerve root, but there is no established standard to judge whether the nerve root tension is restored during the operation, which is crucial for the recovery of neurological function in patients after surgery. This also led to some cases that appeared as satisfactory nerve root decompression during the operation, but nerve dysfunction occurred after the operation.

Although electrophysiological monitoring is widely used in lumbar surgery, there is still a certain incidence of foot drop^3^, ^4^, ^5^, ^6^ postoperatively. This suggests that the cause of foot drop may not be the violent injury to the nerve root during operation, but the chronic ischemia caused by the increase of nerve root tension. If the nerve root tension can be measured immediately and non‐invasively during the operation, it will play a certain role in preventing the occurrence of foot drop and ensuring the recovery of nerve function postoperatively.

At present, there are few studies on nerve root tension. The instrument that can be used to measure nerve root tension during operation has not been invented. This study aimed to investigate the feasibility of intraoperative nerve root tension measurement and to further confirm the correlation between nerve root tension and intervertebral space height. The purpose of this study is summarized as follows: (i) to explore the feasibility of measuring nerve root tension during operation with the measuring device made by the author; (ii) to confirm whether there is an association between nerve root tension and VAS score of patients; (iii) by measuring the nerve root tension at different heights of intervertebral space during operation, a relatively safe intervertebral space stretching range and an appropriate size of intervertebral fusion cage can be provided for clinical surgeons.

## Materials and Methods

### Patient Group

Between June 2020 and August 2021, a total of 54 consecutive patients received posterior lumbar interbody fusion (PLIF) for lumbar disc herniation (LDH) with lumbar spinal stenosis and instability. Among them, there were 33 patients with L4/5 disc herniation and 21 with L5/S1 disc herniation. Inclusion criteria were as follows: (i) huge lumbar disc herniation or simple lumbar disc herniation with lumbar spinal canal stenosis and instability; (ii) leg pain was associated with large LDH; and (iii) failure of conservative treatment over a 3‐month period; (iv) perform PLIF and measure nerve root tension intraoperatively. Exclusion criteria: (i) LDH was associated with lumbar spondylolisthesis, and other lumbar diseases; (ii) cauda equina syndrome.

The average age of the patients at the time of surgery was 54.3 years, ranging from 25 to 68 years. There were 36 males and 18 females.

### Surgical Procedure

All patients underwent PLIF. The patients were treated under general anesthesia in the prone position. After exposure of the dorsal spine at the stenotic level using a standard midline approach, the paraspinal muscles were subperiosteally elevated from the dorsal surface of the spinous process as far as the lateral border of the facet joints. The midline spinous processes, supraspinal and interspinous ligaments, as well as the majority of the lamina were left intact. The decompression was extended to the lateral recess. The transferring nerve roots were well protected using a probe. The nerve root tension was measured before decompression. The posterior longitudinal ligament was incised, the nucleus pulposus tissue was completely removed, and an interbody fusion cage, of appropriate size, was implanted. The pedicle screw system used was EXP (Depuy, America). The pedicle entry point was lateral, at the basis of the transverse process. All screws were inserted under direct vision using a medial angulation appropriate to the spinal level. The pedicle screws were connected with a rod and axially compressed. Before closure, each nerve root was checked to verify adequate decompression and the nerve root tension was measured again. The wound was closed in layers. A deep drain was applied and maintained for a mean duration of 2 days.

### Intraoperative Nerve Root Tension Test

**FIGURE 1:**
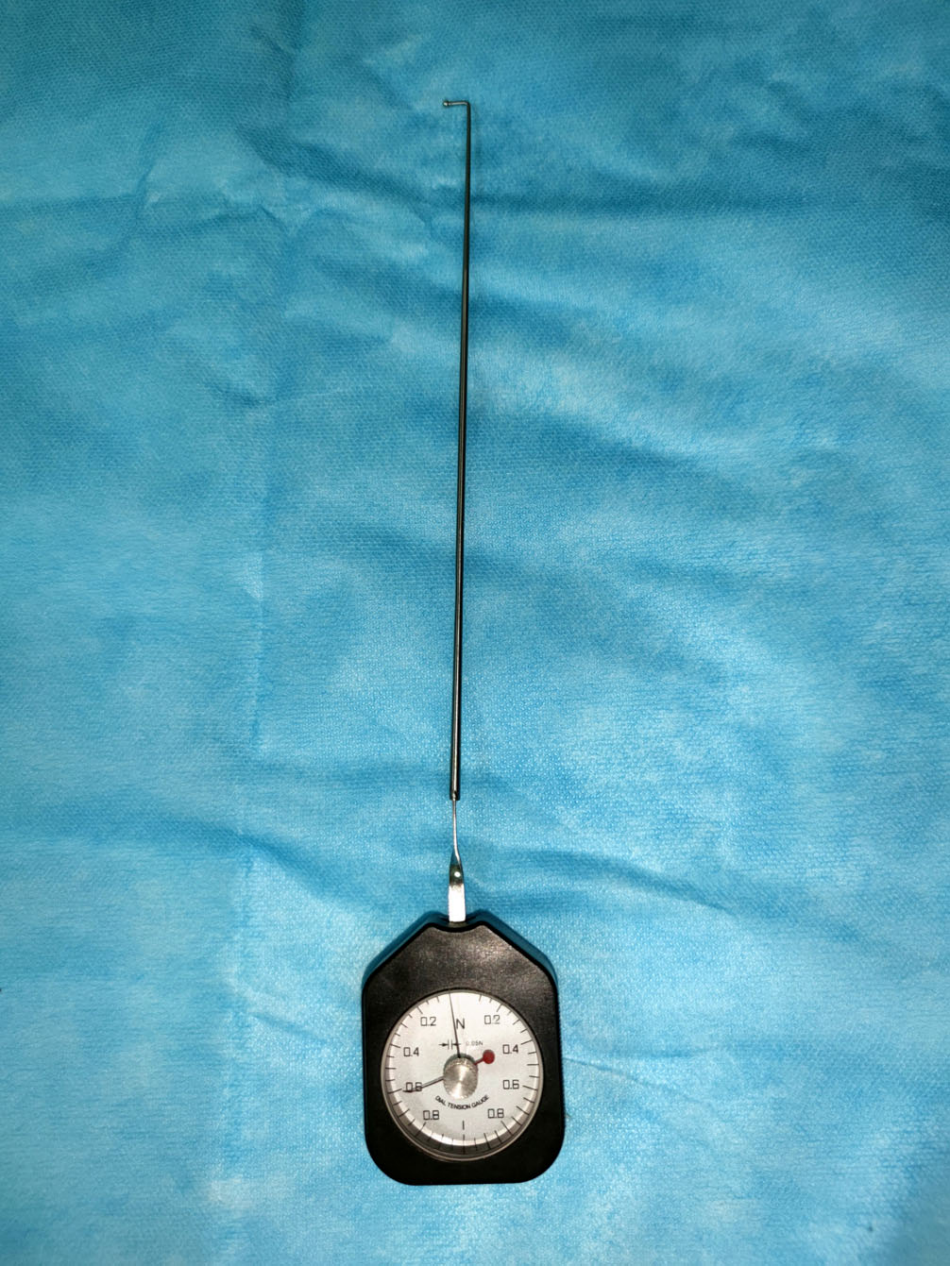
The nerve root tension meter used in this study was modified from the transverse gauge

**FIGURE 2:**
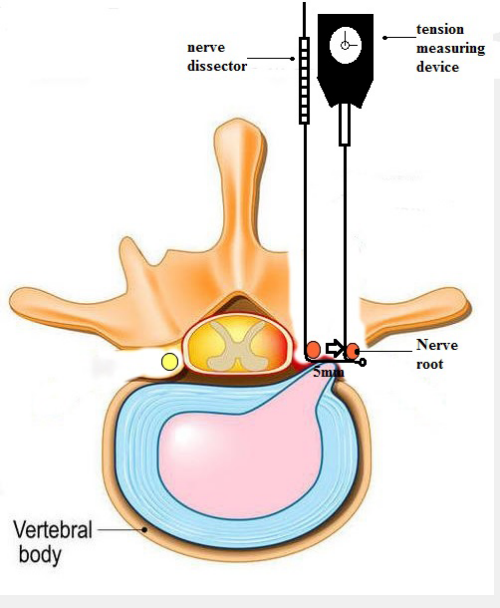
This figure shows how to use the nerve root tension meter during surgery

The nerve root tension measuring device used in this study was modified by the author from a table‐type lateral tension measuring device (height: 59 mm, width: 47 mm, thickness: 19 mm, division value: 0.05 N), and a nerve hook was added to the end of the force‐bearing rod of the original measuring device. The nerve root hook is perpendicular to the pulling direction of the nerve root. Move the nerve root from the static state to the desired position to measure its tension value. The dial of the measuring instrument has a double pointer design (force measuring pointer and memory pointer), which can make operation more convenient and accurate in reading the values (Figure 1). Use the nerve dissector to block the dural sac and expose the L5 and S1 nerve roots during the operation, and the dial counts were read after the nerve roots were pulled through the nerve hook to cause 5 mm displacement according to the length of the hook of the nerve dissector (Figure 2).

**FIGURE 3:**
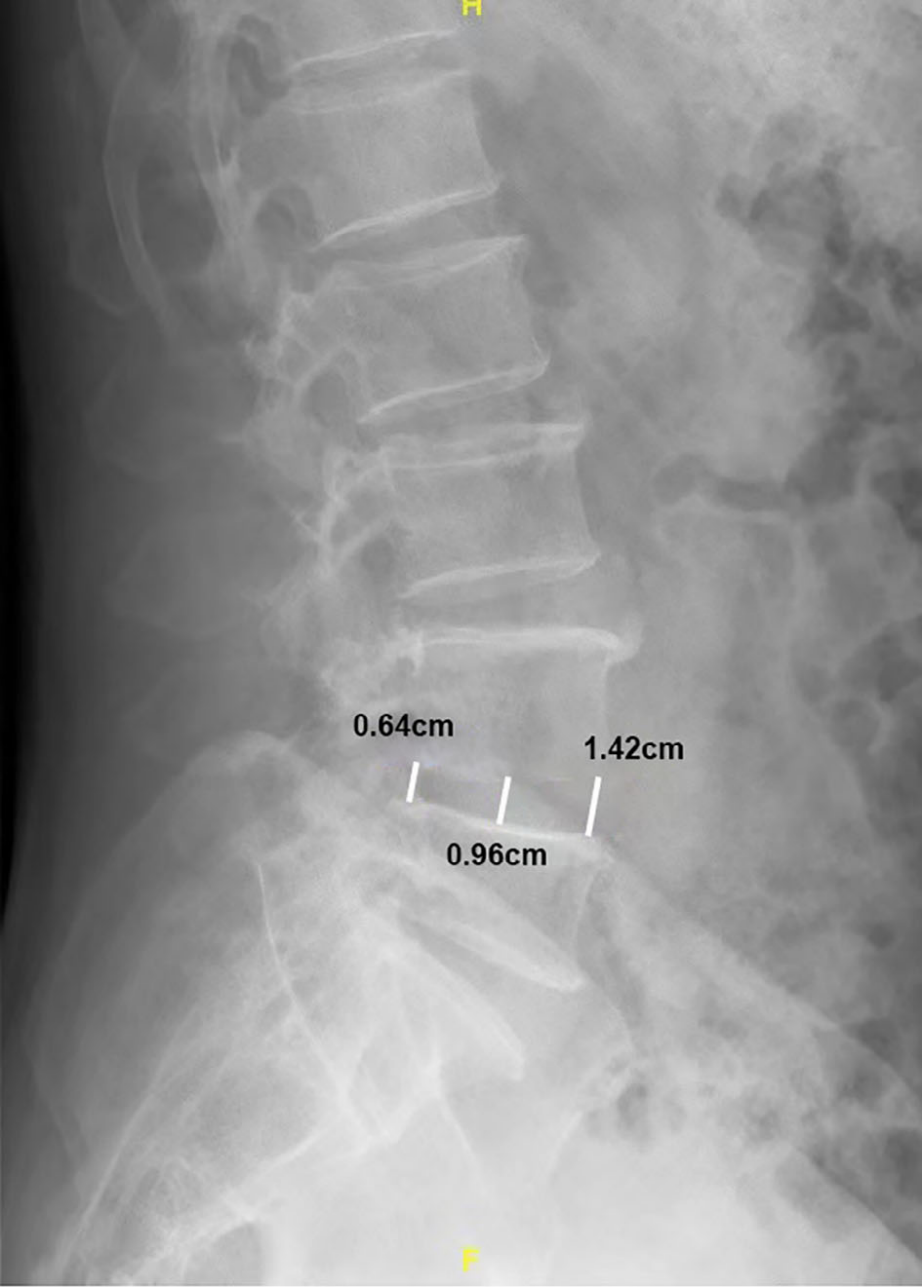
Intervertebral space height measurements were obtained at the anterior edge, center, and posterior edge of each vertebra in the midsagittal plane

The preoperative height of the intervertebral space was measured at the anterior, central, and posterior borders of the diseased intervertebral space in the sagittal plane of the lumbar spine as determined using X‐ray (Figure 3). The average of these data was taken as the value of the intervertebral space height,^2^ and the mean value of intervertebral space height measured preoperatively is set as 100%. The 110%, 120%, 130%, 140% height values were calculated based on preoperative measurements of the intervertebral space height. During the operation, the intervertebral space was stretched to the above heights to measure the nerve root tension. The intraoperative change in nerve root tension was measured by the device. The intervertebral cage model was intraoperatively used to expand the heights after the intervertebral disc was removed. The appropriate intervertebral cage was selected according to the nerve root tension value of the different intervertebral space heights. In order to reduce measurement error, each measurement was performed three times and the mean value was used for statistical analysis. The surgical interventions were approved by the Ethical Committee of the Shanghai Jiao Tong University School of Medicine (SH9H‐2019‐A532‐1). The degree of nerve root traction and the height of intervertebral space stretching during operation are within the normal operation range. Therefore, the measurement of nerve root tension in this study can be considered as noninvasive. So the informed consent was waived by the review board.

### Clinical Outcome Evaluation

Clinical outcome was assessed by means of the visual analog scale (VAS) for leg pain，completed by patients 1 day before operation and 1 day after operation.

### Statistical Analysis

Statistical analyses were performed using SPSS 21.0 (IBM, Armonk, NY, USA). Experimental data are presented as mean ± SD. The Student's *t*‐test was used for the comparison of nerve root tension in different intervertebral space height groups. Linear regression was used to analyze the correlation between VAS score of lower limb pain and nerve root tension at the lesion site. Results were considered statistically significant when the *p*‐value was <0.05.

## Results

### Clinical General Results

A total of 54 patients participated in the study. The average age of the patients at the time of surgery was 54.3 years, ranging from 25 to 68 years. There were 36 males and 18 females. Among them, there were 33 patients with L4/5 disc herniation and 21 with L5/S1 disc herniation.

### Measurement Results of Nerve Root Tension

The preoperative patient intervertebral space height was measured by the sagittal view of the patient's lumbar spine X‐ray: L4/5 10.04 ± 1.2 mm, L5/S1 9.87 ± 1.4 mm. The height of intervertebral space was measured by three doctors, and the mean value of the three groups of data was taken as the analysis data. The consistency of the three groups of data was checked through ICC intra group correlation analysis (ICC = 0.947, *p* < 0.05). Intraoperative measurement of nerve root tension was collected before decompression, after discectomy and distraction to 100%, 110%, 120%, 130%, and 140% of the height of each intervertebral space, and after placement of the cage. The nerve root tension values at 100%, 110%, 120%, and 130% heights were significantly lower than those before decompression, and there was no statistical significance among the four groups. Nerve root tension values were significantly higher at the 140% height and were statistically significant when compared with the 130% height (Table 1).

**TABLE 1:** Nerve root tension values at different intervertebral space heights after intraoperative decompression

Surgical segment	Nerve root tension before decompression (N)	Nerve root tension at different intervertebral space heights after decompression (N)				
		100%	110%	120%	130%	140%
L4/5	1.34 ± 0.18	0.60 ± 0.08^a^	0.60 ± 0.11^b^	0.61 ± 0.15^c^	0.63 ± 0.14^d^	0.84 ± 0.14 ^e^
L5/S1	1.29 ± 0.17	0.61 ± 0.09^a^	0.62 ± 0.10^b^	0.62 ± 0.17^c^	0.64 ± 0.19^d^	0.87 ± 0.17 ^e^

*Note*: Compared with before decompression.

^a^ *p* < 0.01; compared with 100% intervertebral space height.

^b^ *p* > 0.05; compared with 110% intervertebral space height.

^c^ *p* > 0.05; compared with 120% intervertebral space height.

^d^ *p* > 0.05; Gap height comparison.

^e^ *p* < 0.05.

### Clinical Outcomes

The intervertebral cage with an appropriate height was selected according to the nerve root tension value of the different intervertebral space heights. The nerve root tension value was significantly lower than that before decompression (1.32 ± 0.22 N vs. 0.61 ± 0.17 N, *p* < 0.01), and the postoperative VAS score was also significantly improved (7. 0 ± 2.24 vs. 0.8 ± 0.84, *p* < 0.01). The nerve root tension was positively correlated with the VAS score (F = 85.19, *p* < 0.01; F = 78.65, *p* < 0.01). In this study, no patients had postoperative complications, such as foot drop.

## Discussion

In this study, we found: (i) the nerve root tonometry can perform instant noninvasive intraoperative nerve root tension measurement with a self‐made measuring device; (ii) there is a correlation between nerve root tension value and VAS score; (iii) excessive height of intervertebral space will increase the tension value of nerve root. The research purpose is basically realized. It provides a way for clinicians to study nerve root tension, and also provides a new standard for nerve decompression surgery.

### The Relationship between Nerve Root Tension and Nerve Injury

Lumbar radiculopathy is characterized by chronic pain that occurs in the lower back and legs. Early and correct diagnosis is essential. Magnetic resonance imaging is currently the most effective method for diagnosing lumbar radiculopathy.^7^, ^8^, ^9^ However, some patients who have large intervertebral disc herniation according to imaging may have very mild symptoms of pain in the lower back leg. Therefore, González Espinosa de Los Monteros et al.^10^ proposed to check the patient's nerve root status by measuring their neurodynamic or orthopaedic tension. The results of the slump test and Dejerine's triad test combined with the straight leg test and Bragard test in multiple parallel manners showed the highest diagnostic accuracy and validity. The goal of the aforementioned study was to induce pain or paresthesia by increasing tension in the nerve root and determine the incidence of lumbar radiculopathy. This concept is consistent with the theoretical basis of our study: the diagnosis of lumbar radiculopathy should be based on the tension of the nerve root, rather than completely based on imaging technology.

Professor Shi^11^ proposed that the pathological basis of lumbosacral nerve bowstring disease is nerve root or axial traction injury. However, most cases of this disease have no positive imaging findings. Furthermore, Shi's results were consistent with those of our study, that lumbar radiculopathy is caused by increased tension in the nerve root.

Some studies have demonstrated that the pathogenesis of lumbar radiculopathy is due to nerve root axial traction injury. The spinal cord and its nerve roots show rubber‐like elasticity.^12^, ^13^, ^14^ Singh et al.^12^ found that the nerve root is elongated, and the cross‐sectional area is reduced when a nerve root is statically or dynamically pulled.

Electrophysiological monitoring found that the nerve conduction velocity and potential amplitude gradually decreased and the comprehensive action potential area became smaller or even disappeared, which eventually led to complete blockade of nerve root conduction.

Along with the increase in nerve root tension,^13^ stretching tension can not only directly lead to abnormal nerve conduction, but also reduce blood flow in the nerve roots and cause nerve ischemia–reperfusion (I/R) injury. Lin et al.^14^ found that when radiation pain occurred in a straight leg elevation test, the blood flow was reduced by more than 70% in the L5/S1 nerve roots.

Because there is only a thin outer membrane around the nerve root, the nerve root is prone to mechanical injuries and its degree of damage can be high. The resistance to traction only accounts for 10% of the peripheral nerve.^15^, ^16^ In addition, the dorsal root ganglion cells, located on the dorsal side of the nerve root, are not only vulnerable to various inflammatory factors, which cause a series of hypoxia*‐*ischemia reactions, but are also more vulnerable to mechanical injury.^17^, ^18^, ^19^ Ellis et al.^20^ found that mechanical loads that can be easily handled by a healthy nervous system, may be sufficient to aggravate clinical symptoms in patients. Our results also confirmed that, after decompression, the nerve root tension of the patient was significantly reduced, while the VAS score was also significantly improved; these changes were positively correlated.

### Instant Noninvasive Intraoperative Nerve Root Tension Measurement

Methods to protect the nerve roots and avoid excessive traction during surgery are the main concerns for many surgeons. To date, several studies have reported on nerve root blood flow,^14^, ^21^, ^22^ but only a few studies have reported on the mechanical tension in nerve roots, as it is almost impossible to ethically perform such invasive measurements in patients. Only human specimens or in vivo animal models can be used to measure the tension in the spinal nerve roots.^12^, ^23^

Recently, a surgical simulation system was developed, which involves a virtual reality simulator combined with real anatomical structures composed of synthetic materials. An inexperienced doctor can simulate the surgical operation through this system and record the nerve root damage during surgery.^24^ However, the tension value of the nerve root cannot be immediately obtained during surgery, as needed to guide the surgeon in how to decompress properly and expand the intervertebral space to an appropriate height and achieve the best results.

Our device for measuring nerve root tension is modified from a transverse gauge. A nerve hook is installed at the end of the force rod and the tension value is obtained by effective displacement of the nerve root when it is pulled by the nerve hook. The process is the same as with the normal intraoperative traction of the nerve root to expose the intervertebral space, but the degree of traction is much smaller than that of the normal operation. Therefore, it is safer for the nerve root and will not cause additional injury. The surgeon can judge whether decompression is complete by comparing changes in the nerve root tension before and after decompression. When the surgeon installs the intervertebral fusion cage and measures the tension again, a significantly increased tension will indicate that the cage height is too large and needs adjustment.

We consider that the loss of *nerve* root *contractures and elasticity* is in parallel with spinal degeneration and the loss of intervertebral space height in elderly patients. With traditional lumbar spine surgery, the risk of iatrogenic nerve root injury is very high if the nerve root is in the axial direction.

Previous studies have shown that nerve root traction injury caused by excessive expansion of the intervertebral space during surgery and the excessive use of the intervertebral fusion cage will cause nerve root I/R injury, postoperative pain, numbness in the lower limb, and relatively severe complications of foot drop.^5^, ^25^ Professor Shi Jiangang^26^ proposed the three heights (anatomical, natural, and pathological) idea of intervertebral disc. The size of the intervertebral fusion cage should be appropriate to restore the natural height of the intervertebral space to maintain optimal tension of the spinal nerve root, which is the “nerve standard” for spinal decompression. Our study on measuring intraoperative nerve root tension aimed to provide an accurate basis for this standard. The hand‐held nerve root tension meter used in this study belongs to the first‐generation product, which still has some shortcomings, including measurement error caused by unstable control of the tester during the measurement process. Nevertheless, to the best of our knowledge, no similar study has been reported to date. For the first time, the method described in our study enables the immediate and non‐invasive measurement of nerve root tension during surgery, which is a significant new clinical direction.

Only one researcher reported on cage size selection. The study showed that the average height of cages was from 11.25 ± 1.32 mm to 12.38 ± 1.43 mm.^27^ Many spine surgeons chose cage heights by relying on their subjective judgments rather than objective measures. In addition, some doctors have the excessive pursuit of restoring the height of the intervertebral space to result in a supraphysiological intervertebral space, which increases nerve root tension, ischemia and hypoxia injury, and postoperative foot drop complications.

We found that when the height of the intervertebral space was increased to 140% of the original height, there was a significant increase in the risk of injury by nerve root tension. This study provides a reference value for spine surgeons to select interbody fusion cages with the most appropriate height.

### Limitations and Strengths

Of course, because it is an early generation product, some limitations of nerve root tension measuring device are as follows: (i) the instability of the hand‐held measuring device in the measurement process; (ii) the nerve roots above L4 are often located in the ventral side of the dural sac, which makes measurement difficult. The strengths of the study as follows: (i) it is the first time to realize noninvasive measurement of nerve root during operation; (ii) it provides a new method for the study of nerve root tension. In future studies, we will focus on improving the accuracy of the measuring instrument. Meanwhile, this study has highlighted the need for more spine surgeons to pay attention to nerve root tension, to not be satisfied with physical compression of the nerve roots, and not to restore the anatomical height of the intervertebral space.

### Conclusion

This study demonstrates that nerve root tonometry can perform instant noninvasive intraoperative nerve root tension measurement. There is a correlation between nerve root tension value and VAS score. We found that when the height of the intervertebral space was increased to 140% of the original height, the nerve root tension increased the risk of injury significantly.

## Declaration

## Ethics Approval and Consent to Participate

The study protocol was approved by by the Ethical Committee of the Shanghai Jiao Tong University School of Medicine. Informed consent was provided by all participating individuals.

## Consent for Publication

Consent to publish was obtained from all the participants.

## Competing Interests

The authors declare that they have no conflict of interest.

## Author Contributions Statement

Zhiyi Fu: manuscript preparation, literature search, funds collection.

Yujie Wu: study design, data interpretation.

Tong Zhu: data collection, statistical analysis.

## Availability of Data and Material

All relevant data for the research are included in the manuscript.
